# Precise design of nanomedicines: perspectives for cancer treatment

**DOI:** 10.1093/nsr/nwz012

**Published:** 2019-02-05

**Authors:** Jing Wang, Yiye Li, Guangjun Nie, Yuliang Zhao

**Affiliations:** 1 CAS Key Laboratory for Biomedical Effects of Nanomaterials and Nanosafety, CAS Center for Excellence in Nanoscience, National Center for Nanoscience and Technology, China; 2 University of Chinese Academy of Sciences, China

Nanomedicine is a field in which nanomaterials and nanotechnology are applied to improve or create medications. Combining knowledge in various disciplines including nanoscience, biological science, advanced materials and pharmacy, the current interests in nanomedicine mainly focus on exploiting nanomaterials for drug delivery in order to improve the efficacy or safety of conventional treatments (chemical drugs, biological drugs, combination therapies, etc.). In recent years, increasing effort has been directed towards a more ‘precise’ understanding in nanomedicine. This includes not only accurate characterization of existing nanomedicines regarding their intrinsic properties and their biological effects, but also precise design of novel nanomedicines that are able to address diseases in a smart, individualized and safe manner. Taking anti-cancer therapy, one of the hottest topics in nanomedicine research, as an example, we would like to review some recent trends in nanomedicine design, to better recognize the opportunities that may allow us to eventually realize the promise of nanomedicines.

## DRUG LOADING AND DELIVERY

Using nanomaterials to improve the delivery of existing therapeutics represents one of the earliest and most well-established branches of nanomedicine. Loading conventional drug into nanoformulations through encapsulation, conjunction or other techniques may alter their solubility, stability, pharmacokinetics and biodistribution and therefore tune the therapeutic response of the product [1,2]. Since nano-sized particles tend to accumulate in tumors due to the compromised local vascular function, which results in their enhanced permeability retention (EPR) effect, nanocarriers for anti-cancer drugs have drawn particular interest. Several EPR-based nanoformulations for chemotherapeutics, such as Doxil^®^ (PEGylated liposomal doxorubicin) and Abraxane^®^ (albumin-bound paclitaxel), have been approved for clinical use. However, the EPR effect only occurs to a certain extent and was often proved insufficient for these first-generation nanomedicines to achieve the intended efficiency and enhancements in tumor accumulation and biocompatibility [2,3]. Moreover, many other challenges posed by the complex *in vivo* realities, such as blood clearance, tumor heterogeneity and microenvironment barrier, remain unaddressed by their simple nanomedicine design. New types of nanovehicles with additional functions have therefore been developed for controlled release and tumor-targeted delivery, and to incorporate multiple drugs or imaging agents and therapeutics into a single formulation.

**Figure 1.**
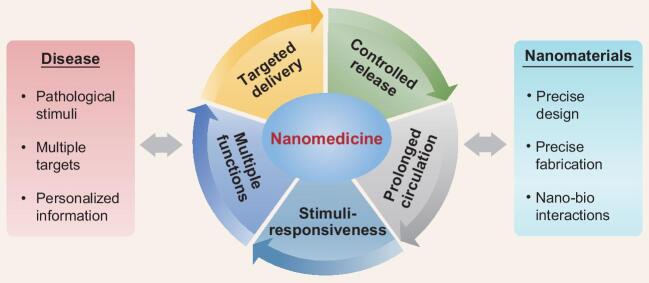
Design of nanomedicine for enhanced therapy. The use of nanosystems could improve the pharmacological properties of existing drugs and create new therapies with multiple targets and functions. Precise design of nanomedicines using advanced nanotechniques according to specific pathological conditions of the patient represents one of the greatest opportunities in this field.

In the rational design of a drug-delivery system, the assistance of nanotechnology could be exploited at multiple levels (Fig. 1). Loading a ‘free drug’ into nanocarriers may lead to changes in its various *in vitro* and *in vivo* pharmacological properties that cannot be satisfactorily tuned in conventional formulations, most typically solubility and stability in the formulation, plasma half-life and biodistribution. Even systems of simple design and low preparation cost, such as liposomes or polymeric particles, could serve to enhance the bioavailability, increase the tolerable dose or reduce the adverse effect of the loaded drug [1,2].

**Figure 2.**
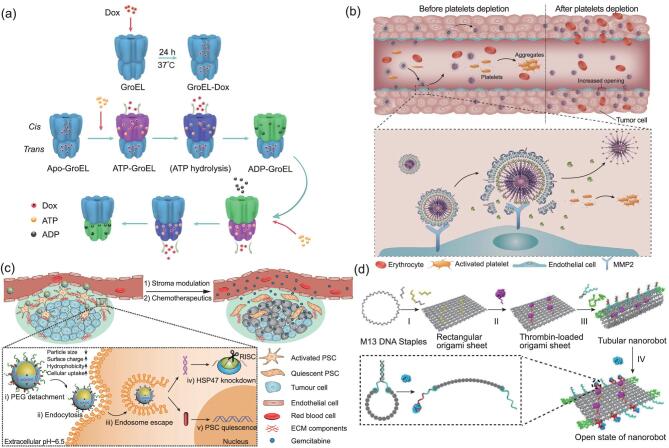
Examples of ‘smart’ anti-cancer nanomedicine design. (a) Nanocage-shaped drug-delivery platform employing a natural protein (GroEL) for delivery and ATP-dependent release of hydrophobic chemotherapeutic (Dox). The drug was released into the tumor tissue in response to the higher ATP level. Reproduced from [5] with permission. Copyright © 2017 American Chemical Society. (b) Lipid–polymer hybrid nanoparticles loaded with anti-platelet antibodies for selective depletion of intratumoral platelets. Eliminating the platelets in tumor vasculature led to the increase in local blood vessel permeability and drug accumulation. Reproduced from [7] with permission. Copyright © 2017 Macmillan Publishers Limited, part of Springer Nature. (c) Polymer-coated gold-nanoparticles loaded with anti-fibrosis drugs for the restoration of desmoplastic stroma in pancreatic cancer. Reproduced from [8] under Creative Commons License http://creativecommons.org/licenses/by/4.0/. (d) DNA origami nanorobot for intravenous administration of thrombin (painted in pink) and selective occlusion of tumor vasculature. Reproduced with permission from [10]. Copyright © 2018 Springer Nature.

However, the capacity of simple nanoparticles in controlling the *in vivo* fate of a drug is quite limited. Therefore, specific features such as surface-targeting ligands, droppable shells, degradable framework or stimuli-responsive components have been integrated into nanosystems to enhance tissue-specific accumulation, prolong blood circulation and enable sustained and disease-triggered release of their cargo. In recent years, progress in nanobiotechnology has further facilitated the design and construction of more complicated nanomaterials, leading to an emerging trend to develop nanosystems as multifunctional therapeutic platforms that combine diverse medications to enhance synergistic effects or combine therapeutics with agents for real-time monitoring or diagnosis. These nanoplatforms could also be made ‘smart’, allowing their targeting, drug-release or degradation behaviors to be guided and/or controlled by particular pathological changes or external signals (e.g. radiation and magnetic field) [2,4]. A recent example is our report of a cage-structured natural-protein-based carrier that selectively release its hydrophobic cargo in response to a high level of ATP (Fig. 2a) [5]. Given the time and expense needed for new drug discovery, such nanomedicines represent a promising approach to improving clinically available therapies with the advantage of extraordinary flexibility of formulation design and the possibility to enter clinical trials in a shorter time. Moreover, with the emergence of precision medicine, they may also facilitate the design of personalized therapies with specific combinations of drugs, targeting ligands and release properties determined by the patient's pathological conditions.

## TARGETING THE MICROENVIRONMENT

Recently, it has been suggested that, in addition to tumor cells, abnormalities related to the tumor microenvironment (TME) could also serve as targets for anti-cancer treatment, and even have several advantages over tumor cells *per se* [6]. These include: (i) TME has an essential role in the growth and invasion of a tumor, and some TME components (e.g. blood vessels or stroma) may be easier to access for drug delivery (since intratumoral penetration was particularly problematic for nanomedicines); (ii) TME provides a wide range of potential targets for delivery, derived from not only its cellular components, but also its extracellular matrix or abiotic aspects such as hypoxia or pH changes; (iii) regulating the TME (e.g. immunotherapy or vessel normalization) does not necessarily rely on highly cytotoxic drugs and may induce less toxicity compared to chemotherapy; (iv) many TME-related changes, such as tissue fibrosis and desmoplasia, are unfavorable factors for radiotherapy or chemotherapy, so TME-targeting pharmaceuticals may also enhance the efficacy of traditional treatments [2,6]. Nanomedicines have been thought to be very useful for TME regulation, not only because they facilitate targeted drug delivery, but also because TME-targeted approaches often require the combination of multiple targets or other therapies that target the tumor cell to generate promising results, opening opportunities for integrated nanosystems. For example, utilizing tumor-targeted nanoparticles to deliver strong platelet inhibitors increased the permeability of tumor vessels without disturbing the systemic coagulation system, and therefore enhanced the efficacy of co-delivered chemotherapeutics (Fig. 2b) [7]. Similar success has been reported in combining two drugs against fibrosis to reverse desmoplasia in pancreatic tumors, which significantly improved the therapeutic output of gemcitabine treatment (Fig. 2c) [8].

## ‘NANO-ONLY’ DRUGS

It has long been noted that the interaction of nanomaterials with biological systems is different from and much more complicated than that of free molecules or bulk-sized materials [9]. Therefore, when administered to the body, the *in vivo* behaviors of a drug-loaded nanosystem are often dominated by factors related to the nanostructure rather than the intrinsic properties of the loaded drug. This implies that, by appropriate design of the nanovehicle, it is possible to direct pharmaceuticals against diseases that are not treatable by their conventional formulations. Typically, a drug may be targeted to particular sites or cells that cannot be sufficiently exposed to the drug when administered freely or be exempted from undesirable biological effects, degradation or clearance so that it could be administered via an unconventional route. Recently, we have shown that, through a smart DNA nanorobot that was only opened by contact with the tumor-associated endothelium, thrombin could be delivered via blood circulation to selectively block tumor blood vessels, avoiding nonspecific thrombotic risk (Fig. 2d) [10]. Being the first work ever to administer thrombin intravenously and to use it against cancer, this work demonstrated that the mission of anti-cancer nanomedicine development is not limited to improving or combining existing therapies, but also includes drawing new ones out of the marriage between pathological knowledge and nanomaterial design.

## PRECISE DESIGN OF NANOMEDICINE IN THE FUTURE

Over the past decades, we have already witnessed enormous advances in nanomaterial science. Future progress in the discovery, fabrication and characterization of nanomaterials would certainly provide more powerful techniques to refine the nanomedicine systems on nanometer or single molecular levels. We believe that such progress would eventually help to perfect the design of nanomedicines and enable more therapies that were not feasible before due to technical limitations. For example, the injectable thrombin formulation mentioned above [10] was only made possible by the precise DNA origami technique that could accurately control the loading site and stimuli-responsive release of this strong coagulation agent.

To further drive nanomedicines towards clinical translation would require comprehensive knowledge of their biological effects. Understanding the interactions at nano–bio interfaces, especially the underlying molecular mechanisms, would be equally critical for the risk assessment of existing nanomedicines and for the creation of novel ones. A series of informative reviews have already documented our current understanding and major knowledge gaps in this area [9,11,12]. An example of how nano–bio interaction studies could promote nanomedicine development may be our investigation of the potential anti-metastasis activity of metallofullerenol Gd@C_82_(OH)_22_, showing that Gd@C_82_(OH)_22_ has specific binding modes with matrix metalloproteinase-9 (MMP-9) in the TME and inhibits the enzyme through an exocite interaction, rendering it more effective than the conventional inhibitors [13]. This demonstrated a new strategy for nanomedicine design that exploits unique nano–bio interactions to overcome the limitations of existing drugs.

Nanomedicine is an emerging interdisciplinary field and the design of smart, effective and safe nanomedicines is still challenging. Although much effort has been dedicated to the development of new nanomedicines, their clinical translation was relatively limited and, even for the most widely used nanostructures, knowledge of their biological effects and *in vivo* course remained flawed. We believe that precise and individualized design of therapeutics according to characteristics of patient and disease represents one of the most important opportunities offered by nanomedicine. However, such a design must be supported by a concrete understanding of diseases and of nano–bio interactions, and by the development of predictive models to guide translational research and risk management; still more effort is needed for such demands. We hope that this brief discussion would help to identify potential directions and gaps towards the next generation of nanomedicines.
